# Early Versus Late Tracheostomy After Decompressive Craniectomy

**DOI:** 10.7759/cureus.3699

**Published:** 2018-12-07

**Authors:** Muhammad Shoaib Safdar Qureshi, Zahid Siddique Shad, Faghia Shoaib, Kamran Munawar, Muhammad Luqman Saeed, Syed Waqar Hussain, Aayesha Qadeer, Muhammad Tariq Khan, Hassan Masood, Azmat Abdullah

**Affiliations:** 1 Internal Medicine, Shifa International Hospital, Islamabad, PAK; 2 Pathology, Bahawal Victoria Hospital, Quaid-E-Azam Medical College, Bahawalpur, PAK; 3 Internal Medicine, Shifa College of Medicine, Islamabad, PAK; 4 Internal Medicine, Khan Research Laboratories Hospital, Islamabad, PAK; 5 Pulmonology, Shifa International Hospital, Islamabad, PAK; 6 Internal Medicine, Shifa International Hospital, Islamabad, USA

**Keywords:** tracheostomy, decompression, craniectomy

## Abstract

Objective

The goal of this study was to determine the efficacy of early tracheostomy (i.e., ≤ 10 days of intubation) compared with a late tracheostomy (> 10 days of intubation) with regards to timing, frequency of ventilator-associated pneumonia (VAP), mortality rate, and hospital stay in patients who received decompressive craniectomy.

Study design

We conducted a retrospective study of data from 168 patients who underwent decompression in the department of critical care medicine at Shifa International Hospital, Islamabad, Pakistan, from January 2017 to December 2017.

Materials and methods

The study included men and women over the age of 18 years who had undergone tracheostomy following decompressive craniectomy in the intensive care unit as a result of stroke, traumatic brain injury, or acute severe injury. Data were analyzed using IBM SPSS Statistics for Windows, Version 23.0 (IBM Corp., Armonk, NY, US). We also applied the Chi-square test, and p ≤ 0.05 was considered significant.

Results

Of 168 patient records reviewed, tracheostomy was performed in 48 patients (21 men, 27 women). In the 48 tracheostomy patients, 15 (31%) were early tracheostomies and 33 (69%) were late tracheostomies. The mean age of patients was 44 ± 11 years. Twenty-eight patients (58.3%) were in the younger age group (age 18 to 45 years) and 20 patients (41.7%) were in the older age group (age > 45 years). Patients who received an early tracheostomy spent significantly less time on a ventilator (≤ 12 days) than those patients receiving a late tracheostomy (> 12 days, p = 0.004). The early tracheostomy group also had a lower incidence rate of VAP than patients with a late tracheostomy (𝑥^2^ = 7.855, p = 0.005). Patients who received an early tracheostomy had lower mortality rates than those who received late tracheostomies (𝑥^2^ = 6.158, p = 0.013). Finally, the length of hospital stay was ≤ 15 days for patients who received early tracheostomies; most patients who received a late tracheostomy had a hospital stay of > 15 days (𝑥^2^ =11.965, p = 0.001).

Conclusions

Performing a tracheostomy within 10 days of intubation following decompressive craniectomy significantly reduced ventilator time, mortality, the incidence of VAP, and length of hospital stay. Given the potential benefits of early tracheostomy in critical care patients following decompressive craniectomy, physicians should consider early tracheostomy in appropriate cases.

## Introduction

Tracheostomy is a surgical procedure mostly used in patients requiring long-term mechanical ventilation [1]. The procedure exteriorizes the trachea to the skin of the neck, resulting in a permanent fistula or opening. The procedure helps prevent tracheal intubation complications (e.g., ventilator-associated pneumonia (VAP), tracheal stenosis, and sinusitis) [2]. Approximately 800,000 acute respiratory patients in the United States undergo tracheostomy each year [3]. North Carolina reported an increased incidence of patients requiring tracheostomy from 284 in 100,000 to 314 in 100,000 from 1996 to 2002 [4]. The average number of tracheostomies performed per year in the United States is over 100,000 [5].

Acute respiratory failure, traumatic or catastrophic neurological insult, and the need for prolonged mechanical ventilation are the most common indications of tracheostomy; upper airway obstruction is the least common indication. In the intensive care unit (ICU), access for mechanical ventilation (prolonged) is the most common reason for tracheostomy. Approximately 10% of patients on mechanical ventilation undergo tracheostomy [6].

The optimal timing of tracheostomy is 10 to 14 days from intubation. Complications of tracheostomy can be categorized into three groups: immediate, early, and late. Immediate complications include hemorrhage, structural damage to the trachea, failure of the procedure, an aspiration event, air embolism, loss of airway, and death [7]. Early complications include hemorrhage, tube displacement, pneumothorax, pneumomediastinum, subcutaneous emphysema, stomal infection, and stomal ulcerations. Late complications include tracheal stenosis, granulation tissue, tracheomalacia, pneumonia, aspiration event, tracheoarterial fistula and tracheoesophageal fistula [8].

McCredie et al. reported that early tracheostomy (defined as occurring ≤ 10 days of intubation) is associated with reduced mechanical ventilation and intensive care unit (ICU) length of stay as compared to late tracheostomy (defined as occurring > 10 days of intubation; p=0.0002). However, early tracheostomy was not associated with short-term mortality reduction (p<0.0001) [9].

Limited data are available on the comparison of early tracheostomy with late tracheostomy in Pakistan. This study aims to determine the efficacy of early tracheostomy (≤ 10 days of intubation) compared with a late tracheostomy (> 10 days of intubation) in terms of timing on a ventilator, frequency of VAP, mortality rate, and length of hospital stay for patients who undergo decompressive craniectomy.

## Materials and methods

We conducted a retrospective review of patient records at the department of critical care medicine at Shifa International Hospital in Islamabad, Pakistan. We reviewed the data of 168 patients over 18 years old who underwent decompression from January 2017 to December 2017. The ethical review board of Shifa International Hospital provided ethical approval for the study. Data were analyzed using IBM SPSS Statistics for Windows, Version 23.0 (IBM Corp., Armonk, NY, US). The mean and standard deviations were calculated for continuous variables while frequency and percentages were calculated for qualitative variables. We used the Chi-square test, and p≤0.05 was considered significant.

## Results

Forty-eight of the 168 patients we reviewed received a tracheostomy; 31% were early while 69% were late. The study data included men and women who had undergone tracheostomy following decompressive craniectomy due to stroke, traumatic brain injury, acute severe injury, and ICU admission. The mean patient age was 44 ± 11 years. Body mass index was ≤ 30 kg/m^2^ in 35 patients (72.9%) and >30 kg/m^2^ in 13 patients (27.1%).

Table 1 presents the association of tracheostomy timing (early vs. late) based on gender and age.

**Table 1:** Association between tracheostomy, gender, and age

Tracheostomy	Gender		Total (%)	Chi-square	P-value
	Male (%)	Female (%)			
Early	6 (12.5%)	9 (18.8%)	15 (31.3%)	0.125	0.72
Late	15 (31.3%)	18 (37.5%)	33 (68.8%)		
Total	21 (43.8%)	27 (56.3%)	48 (100%)		
	Age				
	18–45 years	>45 years			
Early	10 (20.8%)	5 (10.4%)	15 (31.3%)	0.623	0.43
Late	18 (37.5%)	15 (31.3%)	33 (68.8%)		
Total	28 (58.3%)	20 (41.7%)	48 (100%)		

Table 2 presents a comparison of early and late tracheostomy regarding ventilator timing, VAP, mortality, and length of hospital stay.

**Table 2:** Comparison of early versus late tracheostomy in terms of timing, VAP, mortality, and length of hospital stay VAP - ventilator-associated pneumonia

Timing (Ventilator days)	Tracheostomy		Total (%)	Chi-square	P value
	Early (%)	Late (%)			
≤12 days	13 (27.1%)	14 (29.2%)	27 (56.3%)	8.202	0.004
>12 days	2 (4.2%)	19 (39.6%)	21 (43.8%)		
VAP					
No	12 (25%)	12 (25%)	24 (50%)	7.855	0.005
Yes	3 (6.3%)	21 (43.8%)	24 (50%)		
Mortality					
No	10 (20.8%)	31 (64.6%)	41 (85.4%)	6.158	0.013
Yes	5 (10.4%)	2 (4.2%)	7 (14.6%)		
Length of hospital stay					
≤15 days	15 (31.3%)	16 (33.3%)	31 (64.6%)	11.965	0.001
>15 days	0 (0%)	17 (35.4%)	17 (35.4%)		
Total	15 (31.3%)	33 (68.8%)	48 (100%)		

**Figure 1:**
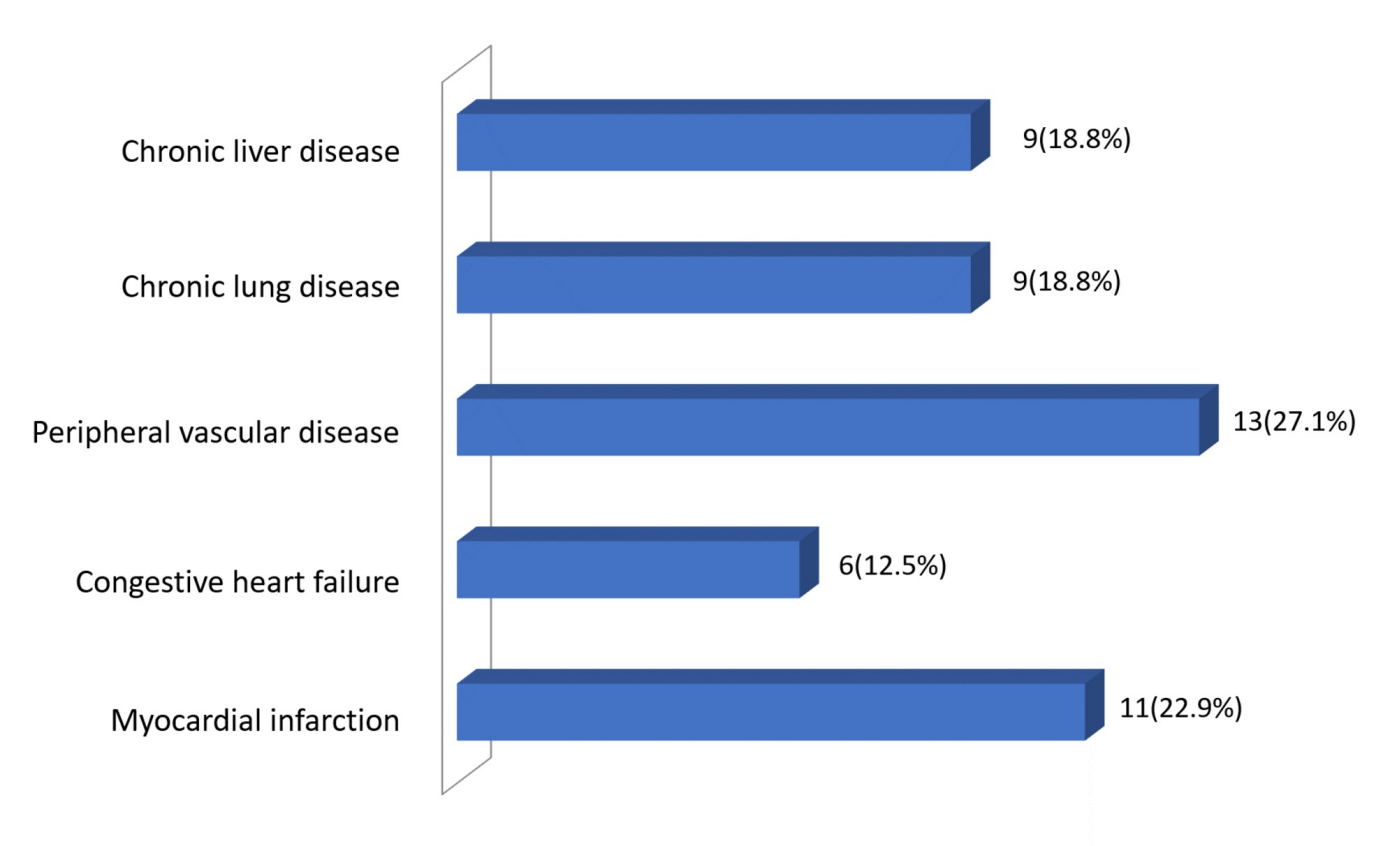
Patient disease distribution data

Figure 1 presents patient disease distribution data.

## Discussion

Patients requiring tracheostomy after decompressive surgery always represent a challenge for physicians [10]. Ranes et al. suggested tracheostomies can be divided into two categories, early and late. Their study of tracheostomies had 1:1 randomization and the comparison is somewhat conflicting (≤ 12 days versus > 12 days, p=0.004) [11].

In our study, the time spent on a ventilator in days was significantly lower in those receiving an early tracheostomy as compared with ventilator time for patients receiving a late tracheostomy (≤ 12 days versus > 12 days, p=0.004). Holzapfel et al. reported divergent results on mechanical ventilation time and found no difference in ventilator time between early and late tracheostomy [12]. However, Cavaliere et al. reported a shorter duration of ventilator time in their early tracheostomy group with a high probability of discharge from ICU on the 28th day [13].

While we found a lower incidence of VAP in the early tracheostomy group as compared to the late tracheostomy group, Heffner et al. reported contradictory findings of a higher incidence of VAP in early tracheostomy patients (55%) rather than late tracheostomy patients [14].

We found a lower mortality rate associated with early tracheostomy rather than late tracheostomy. Nieszkowska et al. reported that early tracheostomies reduced long-term mortality in brain injuries [15]. However, Diehl et al. reported that a late tracheostomy was associated with a reduction in short-term mortality [16].

While our results indicate that an early tracheostomy is associated with a shorter hospital stay, Francois et al. found no significant difference in the duration of hospital stay between early and late tracheostomy [17]. A similar study reported that early tracheostomy was associated with a reduced hospital stay for patients in the ICU (5%) compared to a late tracheostomy (p=0.001) [18].

Our study was limited in that reviewing data from a single center limits the generalizability of our study findings. Further studies with larger patient populations are warranted to confirm our results.

## Conclusions

Performing early tracheostomy following decompressive craniectomy had a significant impact on reducing the ventilator use duration, mortality, VAP, and hospital stay compared to late tracheostomy. Given the potential benefits of early tracheostomy in critical care patients following decompressive craniectomy, physicians should consider early tracheostomy in appropriate circumstances.
